# Description of Dynamic Recrystallization Behaviors and Grain Evolution Mechanisms during the Hot Forming Process for SAE 5137H Steel

**DOI:** 10.3390/ma15165593

**Published:** 2022-08-15

**Authors:** Yu-Qing Zhang, Guo-Zheng Quan, Sheng Lei, Jiang Zhao, Wei Xiong

**Affiliations:** 1 Chongqing Key Laboratory of Advanced Mold Intelligent Manufacturing, School of Material Science and Engineering, Chongqing University, Chongqing 400044, China; 2 State Key Laboratory of Materials Processing and Die & Mould Technology, Huazhong University of Science and Technology, Wuhan 430074, China; 3 Key Laboratory of Advanced Reactor Engineering and Safety of Ministry of Education, Collaborative Innovation Center of Advanced Nuclear Energy Technology, Institute of Nuclear and New Energy Technology, Tsinghua University, Beijing 100084, China

**Keywords:** SAE 5137H steel, dynamic recrystallization, DRX kinetics model, cellular automaton, modeling and simulation

## Abstract

Describing the dynamic recrystallization (DRX) behaviors and grain evolution mechanisms in the hot forming process contributes to controlling microstructures and enhancing mechanical properties of materials. Here, the isothermal compression experiments for SAE 5137H steel were conducted under temperatures of 1123–1483 K and strain rates of 0.01–10 s^−1^. The DRX kinetics models, including DRX volume fraction and grain size models, and the meso-scale cellular automaton (CA) models, were established based on the obtained true stress–strain curves and microstructure observation results. In order to dynamically reveal DRX behaviors and grain morphology evolution, a multi-field and multi-scale coupling finite element (FE) model for the hot compression process was developed by embedding the solved DRX kinetics models and CA models. Results show that the DRX volume fraction and grain size increase with temperature increasing and strain rate decreasing. The DRX grains are easier to nucleate at the initial grain boundaries. As strain increases, DRX grains grow up by devouring the matrix grains until DRX occurs completely. The microstructures after compression are composed of equiaxed DRX grains. Finally, the comparisons of grain size between experimental results and simulation results were performed. The mean relative errors between experimental results and predicted results from DRX kinetics models, and between experimental results and predicted results from CA models, were evaluated as 6.5% and 6.0%, respectively. It proves that the developed FE model can well describe the microstructure evolution in the hot deformation process of SAE 5137H steel.

## 1. Introduction

SAE 5137H, as a medium-carbon alloy steel, is widely applied in automobiles, railways, and ships due to its superior toughness and high strength [1,2]. The hot forging method is frequently used for fabricating the components with SAE 5137H steel. During a hot forming process, three deformation mechanisms, i.e., work hardening (WH), dynamic recovery (DRV) softening, and dynamic recrystallization (DRX) softening coexist, which directly determine the microstructure evolution [3,4,5]. As for DRX, it is the dominant grain refinement mechanism, and the microstructures with finer grains contribute to improving the comprehensive properties of materials [6,7]. It is well-accepted that the DRX behaviors are sensitively affected by temperature, strain, and strain rate, and show a complicated nonlinear relationship with the three deformation parameters [8,9,10]. Hence, it is of great significance to accurately describe the DRX behaviors and grain evolution mechanisms for controlling the microstructures in the hot forming process of SAE 5137H steel.

Up to now, much research has been carried out on developing DRX kinetics models to describe the correspondence between DRX behaviors and deformation parameters, and furthermore these models were implanted into finite element (FE) models to predict the dynamic evolution of microstructures in the hot forming process of materials. Quan et al. [9] established the DRX volume fraction model and grain size model of Nimonic 80A superalloy based on the isothermal compression tests, and implanted the obtained DRX kinetics models into an FE platform to predict grain size evolution. In the same way, Chen et al. [3] and Lin et al. [11] constructed the DRX mathematical models of 42CrMo steel, and discussed the effects of deformation temperature and strain rate on DRX volume fraction evolution during a hot upsetting process. Li et al. [12] and Wan et al. [13] also predicted the evolution of DRX volume fraction in the hot compression process for a micro-alloyed plastic mold steel (MnCrNb) and TiAl-based alloy by combining DRX kinetics models with FE simulation, respectively. Although the developed DRX kinetics models are capable of predicting the evolution of grain size and DRX volume fraction, the grain morphology cannot be reconstructed to actually describe the detailed grain evolution mechanisms. 

With the rapid development of image simulation technology, the cellular automaton (CA) method has been extensively applied in the topographic simulation of microstructures to uncover the meso-scale grain evolution mechanisms in DRX, DRV, grain growth, etc. In 1998, Goetz et al. [14] simulated the grain morphology evolution in the DRX process by the CA method. Liu et al. [15,16] analyzed the DRX behaviors in the hot compression process of a Ni-based superalloy by a CA model. Chen et al. [17] simulated the influence of initial grain size on the DRX behaviors of 42CrMo steel in the hot compression process by an extended CA model. Ding et al. [18] constructed a new model that couples fundamental metallurgical principles of DRX with the CA method, so as to simulate the plastic flow behaviors of an oxygen-free high-conductivity (OFHC) copper and its microstructure evolution. Li et al. [19] developed a 3D cellular automata-crystal plasticity FE model, by which the multi-scale interactions among heterogeneous deformation, DRX microstructure evolution, and mechanical responses in titanium alloys were predicted. So far, it has not been reported in describing the DRX behaviors and grain evolution mechanisms during the hot forming process for SAE 5137H steel. 

This work aims to uncover the DRX behaviors and grain evolution mechanisms in the hot forming process of SAE 5137H steel from the microscopic scale and mesoscopic scale. Here, a series of isothermal compression experiments were conducted over the deformation temperature range of 1123–1483 K and the strain rate range of 0.01–10 s^−1^. Based on the results of compression experiments and microstructure observation, the DRX kinetics models including DRX volume fraction and grain size models, and the meso-scale CA model were constructed. Then, these solved models were embedded into a hot compression FE model to numerically describe the microstructure evolution in the hot deformation process of SAE 5137H steel. The established DRX kinetics models revealed the distributions of DRX volume fraction and grain size, and the meso-scale CA models uncovered the evolution process of grain morphology. Finally, the comparisons of grain size between the experimental and simulation results indicate that the simulation results have a great agreement with experimental ones. This work contributes to the design and optimization of processing parameters in the hot forming process of SAE 5137H steel for obtaining the desired microstructures. 

## 2. Experiment Procedure

**Figure 1:**
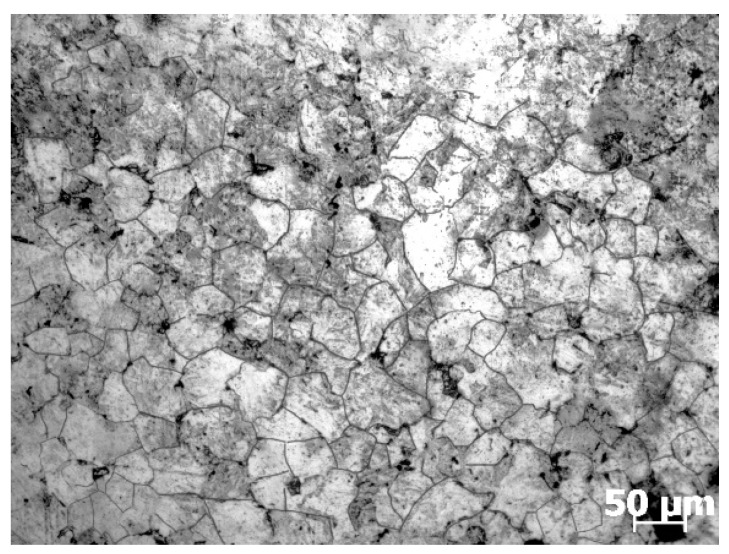
Initial microstructure of the as-received SAE 5137H steel without deformation.

**Figure 2:**
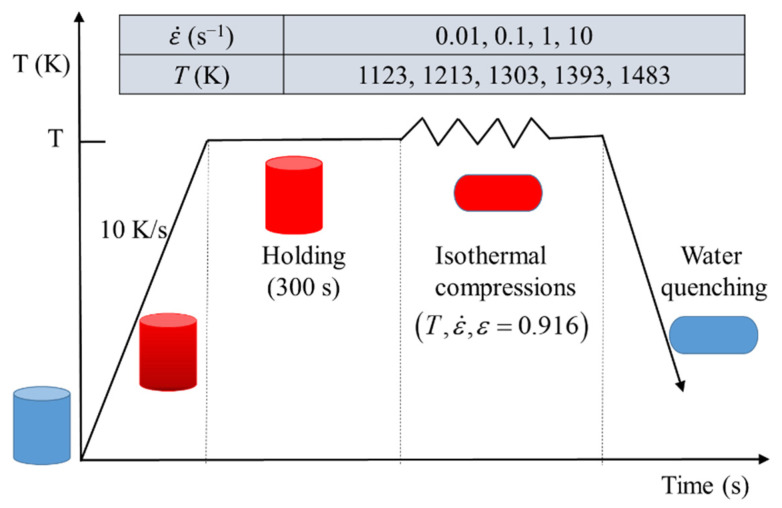
Processing routes of the isothermal compression test.

The studied material in the present work was SAE 5137H steel whose chemical composition was given in Table 1. The initial microstructure with an average grain size of 64.8 μm was exhibited in Figure 1. Twenty standard cylindrical specimens with the dimension of *Φ*10 mm × 12 mm were cut from a rolled billet for isothermal compression tests conducted on a Gleeble 3500 thermal simulator. These tests were performed under five different deformation temperatures of 1123 K, 1213 K, 1303 K, 1393 K, and 1483 K, and four diverse strain rates of 0.01 s^−1^, 0.1 s^−1^, 1 s^−1^, and 10 s^−1^. The processing routes were simplified as Figure 2. In each isothermal compression test, the specimen was electrically heated to a specified temperature with a heating rate of 10 K/s, and then it was held at this temperature for 300 s. Subsequently, the heated specimen was compressed up to a height reduction of 60% (true strain of 0.916) with the proposed strain rate. After that, the deformed specimen was immersed in water immediately to retain the elevated temperature microstructures. 

**Figure 3:**
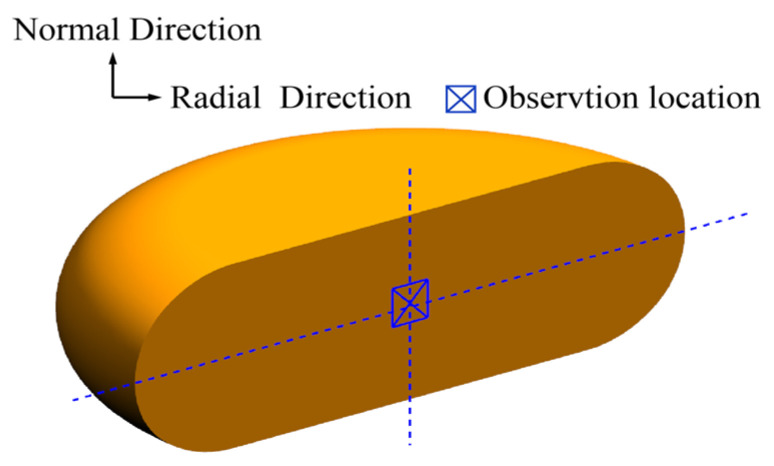
Schematic illustration of the section after an isothermal compression test.

In order to characterize the deformed microstructures, the compressed specimens were cut in half along their compression axials, and the schematic illustration of the section was shown in Figure 3. The cutting surfaces were polished mechanically firstly, and then the chemical corroded with a solution of 50 mL distilled water, 2 g picric acid, and 2 g detergent at the temperature of 333 K for 5–10 min. Finally, the microstructures in the center area of the cutting surfaces were characterized by metallographic microscope, as marked “X” in Figure 3. The average grain size of microstructures was statistically analyzed using the intercept method in Image-Pro Plus 6.0 software 

## 3. Description of DRX Behaviors and Kinetics

### 3.1. DRX Characteristics in Stress–Strain Curves

**Figure 4:**
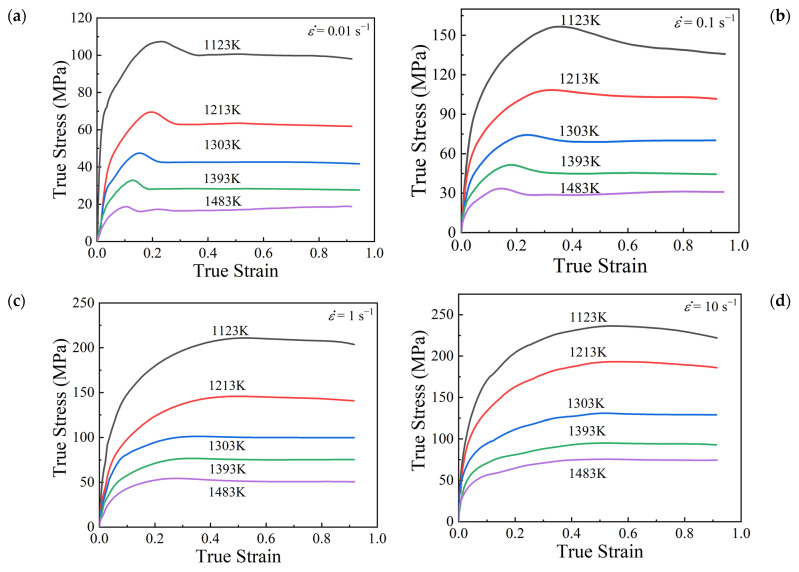
True stress–strain curves of SAE 5137H steel under temperatures of 1123–1483 K and different strain rates: (**a**) 0.01 s^−1^, (**b**) 0.1 s^−1^, (**c**) 1 s^−1^, (**d**) 10 s^−1^.

Figure 4 illustrates the obtained true stress–strain curves of SAE 5137H steel from isothermal compression experiments. According to Figure 4, it is obvious that the true stress–strain curves show the evident features of DRX softening behaviors. The variation of flow stress with true strain can be divided into three stages. At the beginning of deformation, flow stress increases rapidly with increasing true strain due to the effect of WH and accordingly accumulation of dislocation. In the second stage, when the accumulated dislocation density exceeds a threshold, dynamic recovery (DRV) and dynamic recrystallization (DRX) occur and then accelerate the annihilation of dislocation. However, since DRV and DRX are too weak to offset WH, the flow stress still increases with a relatively low increasing rate. When the flow stress continuously increases to a peak value, work hardening and dynamic softening behaviors reach a balance at the first time. In the third stage, DRX softening mechanism dominates, and the flow stress begins to decrease slowly with the continuous increase of strain. Finally, the flow stress tends to be stable owing to the dynamic equilibrium between WH and DRX softening behaviors. Moreover, it also can be summarized from Figure 4 that deformation temperature and strain rate have significant influence on the flow stress. At higher temperatures and lower strain rates, the flow stress is relatively lower. These phenomena are due to the fact that there is sufficient time for the energy accumulation and the nucleation of recrystallized gains at a lower strain rate. In addition, higher temperature accelerates the occurrence of dislocation motion and the migration of grain boundaries [9,20,21]. 

### 3.2. Modeling of DRX Kinetics

#### 3.2.1. Initiation of DRX $\varepsilon_{c}$

**Figure 5:**
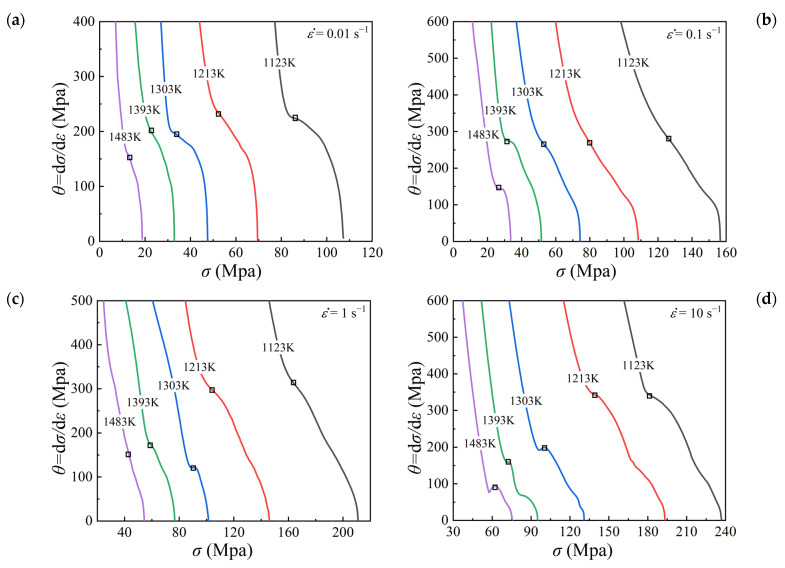
Curves of θ−σ under temperatures of 1123–1483 K and different strain rates: (**a**) 0.01 s^−1^, (**b**) 0.1 s^−1^, (**c**) 1 s^−1^, (**d**) 10 s^−1^. The marked squares represent the inflection points which correspond to the critical condition for the initiation of DRX.

Usually, the critical strain $\varepsilon_{c}$ representing the onset of DRX can be derived from the strain hardening rate curves [22,23]. By taking the derivative of each true stress–strain curve, the strain hardening rate θ=dσ/dε was calculated, and the θ−σ curves for all the studied deformation conditions were plotted in Figure 5. The inflection points as marked by the squares in Figure 5 correspond to the critical condition for the initiation of DRX [24]. Then, the critical stress was obtained from the θ−σ curves, and the critical strain $\varepsilon_{c}$ was identified from the true stress–strain curves accordingly.

The critical strain $\varepsilon_{c}$ is linearly related to peak strain $\varepsilon_{p}$, and its relationship can be described as Equation (1) [9,25]:

(1) $$\varepsilon_{c}=a\varepsilon_{p}$$

where *a* is a material constant. The peak strain is the function of deformation activation energy $Q_{1}$, deformation temperature *T*, strain rate $\dot{\varepsilon }$ and the initial grain size $d_{0}$, as Equation (2):

(2) $$\varepsilon_{p}=a_{1}d_{0}^{n_{1}}{\dot{\varepsilon }}^{m_{1}}\exp\left(Q_{1}/RT\right)$$

where *R* is the universal gas constant taken as 8.31 J mol^−1^ K^−1^; *a*_1_, *n*_1_ and *m*_1_ are material constants. As the effect of initial grain size has been overlooked in this work, i.e., *n*_1_ = 0, Equation (2) can be replaced by Equation (3):

(3) $$\varepsilon_{p}=a_{1}{\dot{\varepsilon }}^{m_{1}}\exp\left(Q_{1}/RT\right)$$

**Figure 6:**
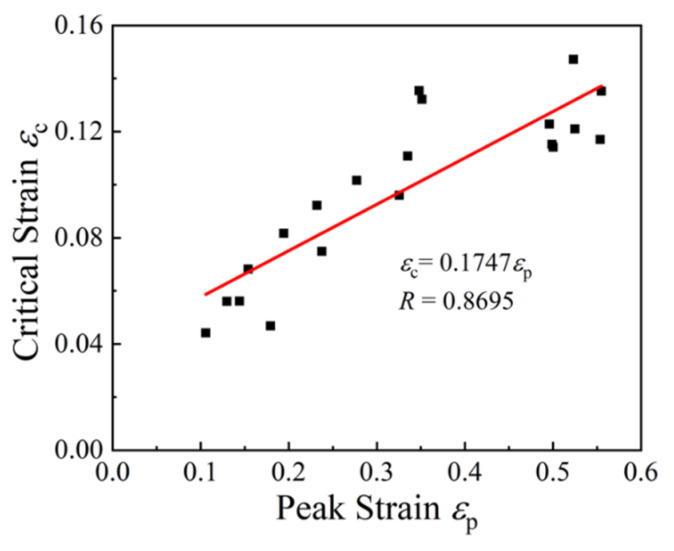
Relationship of $\varepsilon_{c}$~$\varepsilon_{p}$.

According to the true stress–strain curves (Figure 4) and the strain hardening rate curves (Figure 5), the values of peak strain and critical strain corresponding to diverse temperatures and strain rates were obtained, as given in Table 2. According to Equation (1), the relationship between $\varepsilon_{c}$ and $\varepsilon_{p}$ was fitted linearly with a correlation coefficient of 0.8695, as depicted in Figure 6. The slope a was calculated as 0.174. 

Take a natural logarithm on both sides of Equation (3) and obtain Equation (4):

(4) $$\ln\varepsilon_{p}=\ln a_{1}+m_{1}\ln\dot{\varepsilon }+Q_{1}/RT$$

**Figure 7:**
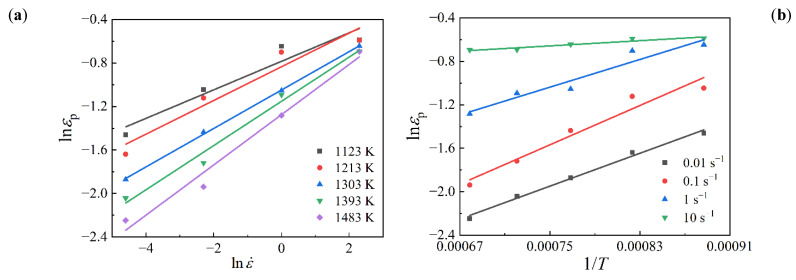
Relationships of (**a**) $\ln\varepsilon_{p}$~$\ln\dot{\varepsilon }$ and (**b**) $\ln\varepsilon_{p}$ ~1/T.

From Equation (4), it can be derived that $m_{1}=\partial \ln\varepsilon_{p}/\partial \ln\dot{\varepsilon }$ and $Q_{1}=R\partial ln\varepsilon_{p}/\partial (1/T)$. The relationships of $\ln\varepsilon_{p}$~$\ln\dot{\varepsilon }$ and $\ln\varepsilon_{p}$~1/T were fitted linearly as Figure 7. Then, the average values of *m*_1_ and $Q_{1}$ were calculated as 0.1791 and 24201.59, respectively. By substituting the average values of *m*_1_ and $Q_{1}$ into Equation (3), the value of *a*_1_ was calculated as 0.03811. Therefore, the models of critical strain for SAE 5137H steel were obtained as Equations (5) and (6):

(5) $$\varepsilon_{c}=0.1747\varepsilon_{p}$$

(6) $$\varepsilon_{p}=0.03811{\dot{\varepsilon }}^{0.1791}\exp\left(24201.59/RT\right)$$

#### 3.2.2. The Kinetics of DRX

Here, the Johnson–Mehl–Avrami–Kolmogorov (JMAK) equation was employed to describe the evolution of DRX volume fraction in the hot forming process for SAE 5137H steel, and its formulation can be expressed as Equation (7) [5,26]:

(7) $$X_{\mathrm{drx}}=1-\exp\left[-\beta_{d}{\left(\frac{\varepsilon -\varepsilon_{c}}{\varepsilon_{0.5}}\right)}^{k_{d}}\right]$$

where $X_{\mathrm{drx}}$ is DRX volume fraction; $\beta_{d}$ and $k_{d}$ are material constants; $\varepsilon_{0.5}$ is the strain for 50% volume fraction of DRX, which can be calculated from Equation (8) [3]:

(8) $$\varepsilon_{0.5}=a_{2}d_{0}{}^{n_{2}}{\dot{\varepsilon }}^{m_{2}}\exp(Q_{2}/RT)$$

where *a*_2_, *n*_2_, and *m*_2_ are material constants; *Q*_2_ is the activation energy for recrystallization. 

**Figure 8:**
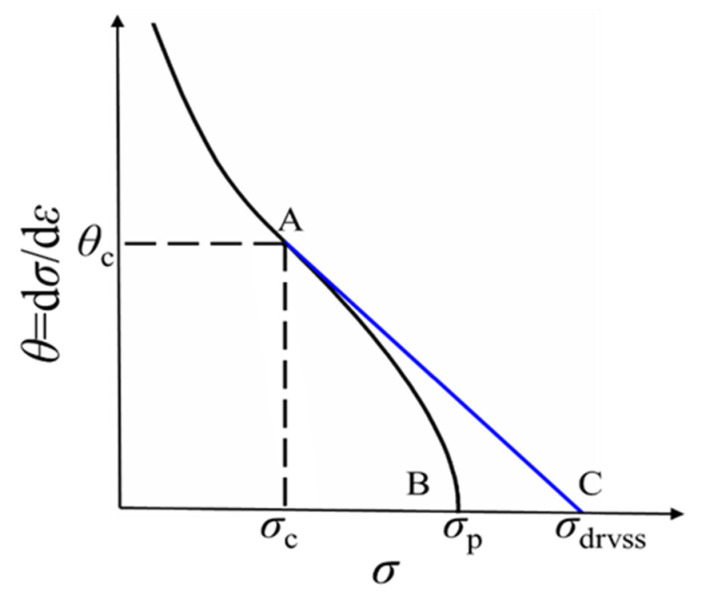
Plot of θ−σ curve employed to determine $\sigma_{\mathrm{drvss}}$ by the intercept with the horizontal axis. The point A represents the critical point for the initiation of DRX; point B represents the point of peak stress; and point C represents the point of steady state stress in the ideal DRV-type stress–strain curves.

It is widely accepted that the DRX volume fraction can also be evaluated from true stress–strain curves, and its calculation formula can be described as Equation (9) [25,27]:

(9) $$X_{\mathrm{drx}}=\frac{{\left(\sigma_{\mathrm{drvx}}\right)}^{2}-{\left(\sigma_{\mathrm{drxx}}\right)}^{2}}{{\left(\sigma_{\mathrm{drvss}}\right)}^{2}-{\left(\sigma_{\mathrm{drxss}}\right)}^{2}}$$

where $\sigma_{\mathrm{drvss}}$ and $\sigma_{\mathrm{drvx}}$ are the steady state stress and transient state stress in the ideal DRV-type stress–strain curves, respectively; $\sigma_{\mathrm{drxss}}$ and $\sigma_{\mathrm{drxx}}$ are the steady state stress and transient state stress in the ideal DRX-type stress–strain curves, respectively. The values of $\sigma_{\mathrm{drvss}}$ and $\sigma_{\mathrm{drvx}}$ can be calculated by the supposed θ−σ curve without DRX, which is shown by the blue line in Figure 8. In Figure 8, point A represents the critical point for the initiation of DRX; point B represents the point of peak stress; and point C represents the point of steady state stress in the ideal DRV-type stress–strain curves. The supposed curve in Figure 8 shows that the work hardening rate θ decreases linearly to zero with the flow stress increasing from the critical stress $\sigma_{c}$ to the peak stress $\sigma_{p}$, and it can be represented as Equation (10):

(10) θ=dσ(ε)/dε=kσ(ε)+b

**Figure 9:**
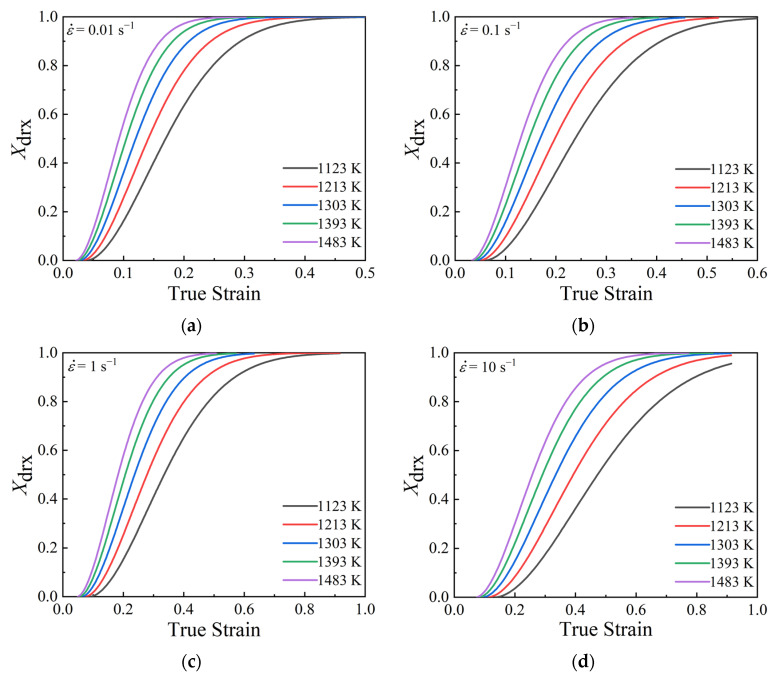
Curves of calculated $X_{\mathrm{drx}}$ under temperatures of 1123–1483 K and different strain rates: (**a**) 0.01 s^−1^, (**b**) 0.1 s^−1^, (**c**) 1 s^−1^, (**d**) 10 s^−1^.

By combining the critical stress $\sigma_{c}$ and its corresponding work hardening rate $\theta_{c}$ with the slope of θ−σ curve at the critical point A, the supposed θ−σ curve and the values of $\sigma_{\mathrm{drvss}}$ and $\sigma_{\mathrm{drvx}}$ can be obtained. As for the values of $\sigma_{\mathrm{drxx}}$ and $\sigma_{\mathrm{drxss}}$, they can be derived directly from the obtained true stress–strain curves. Therefore, the values of $X_{\mathrm{drx}}$ under different temperatures, strain rates, and true strains can be determined. Figure 9 illustrates the curves of calculated $X_{\mathrm{drx}}$ under temperatures of 1123–1483 K and strain rates of 0.01–10 s^−1^.

Take natural logarithms on both sides of Equation (8) and obtain as Equation (11):

(11) $$\ln\varepsilon_{0.5}=\ln a_{2}+n_{2}\ln d_{0}+m_{2}\ln\dot{\varepsilon }+Q_{2}/RT$$

**Figure 10:**
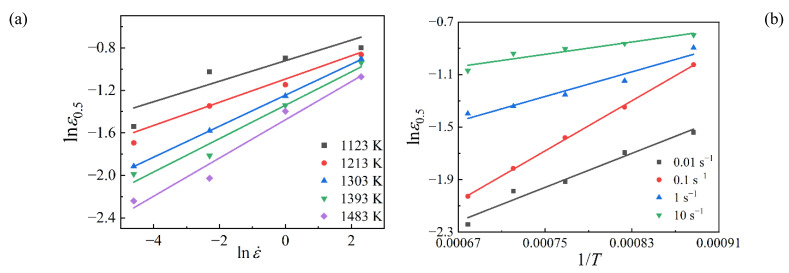
Relationships of (**a**) $\ln\varepsilon_{0.5}$~$\ln\dot{\varepsilon }$ and (**b**) $\ln\varepsilon_{0.5}$ ~1/T.

As the effect of initial grain size has been overlooked in this work, i.e., *n*_2_ = 0, it can be easily obtained from Equation (11) that $m_{2}=\partial \ln\varepsilon_{0.5}/\partial \ln\dot{\varepsilon }$ and $Q_{2}=R\partial ln\varepsilon_{0.5}/\partial (1/T)$. The relationships of $\ln\varepsilon_{0.5}$~$\ln\dot{\varepsilon }$ and $\ln\varepsilon_{0.5}$~1/T were fitted linearly as Figure 10. Then, the material constants *m*_2_ and *Q*_2_ were evaluated as 0.1376 and 23,059.65, respectively. By substituting the calculated results of *m*_2_ and *Q*_2_ into Equation (8), the value of *a*_2_ was calculated as 0.03432. 

Taking a natural logarithm on both sides of Equation (7) yields Equation (12):

(12) $$\ln\left[-\ln(1-X_{\mathrm{drx}})\right]\varepsilon_{0.5}=\ln\beta_{d}+k_{d}\ln\left[\left(\varepsilon -\varepsilon_{c}\right)/\varepsilon_{0.5}\right]$$

**Figure 11:**
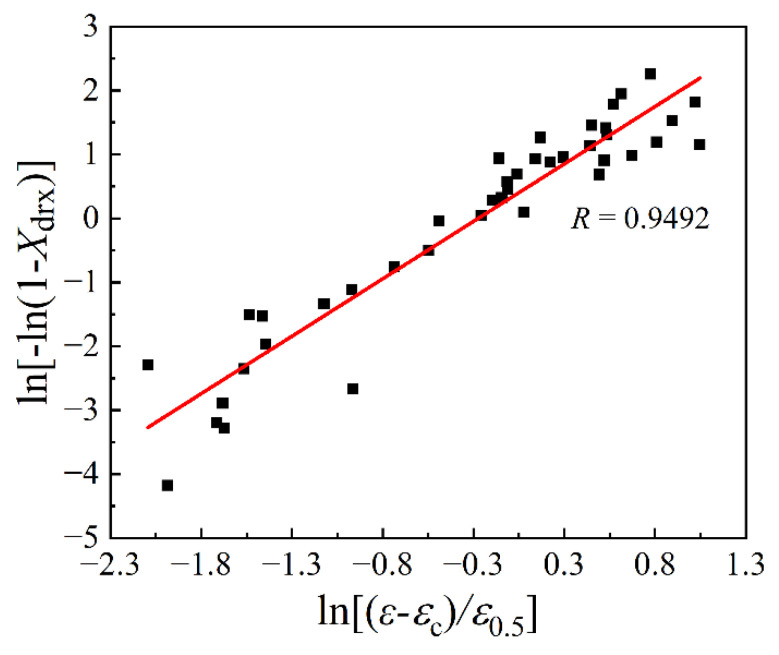
Relationship of $\ln\left[-\ln(1-X_{\mathrm{drx}})\right]$~$\ln\left[\left(\varepsilon -\varepsilon_{c}\right)/\varepsilon_{0.5}\right]$.

Based on Equation (12), it is derived that $k_{d}=\partial \ln\left[-\ln(1-X_{\mathrm{drx}})\right]/\partial \ln\left[\left(\varepsilon -\varepsilon_{c}\right)/\varepsilon_{0.5}\right]$. From Figure 9, the values of $X_{\mathrm{drx}}$ corresponding to the true strains of 0.2, 0.5, and 0.8 for various deformation temperatures and strain rates were determined. The relationship of $\ln\left[-\ln(1-X_{\mathrm{drx}})\right]$~$\ln\left[\left(\varepsilon -\varepsilon_{c}\right)/\varepsilon_{0.5}\right]$ was fitted linearly with a correlation coefficient of 0.9492, as demonstrated in Figure 11. In addition, the slope and intercept of the fitted line that equals the values of $k_{d}$ and $\ln\beta_{d}$, were calculated as 1.7959 and 1.69, respectively.

Therefore, the DRX kinetics models for SAE 5137H steel can be summarized as Equation (13):

(13) $$\left\{\begin{array}{l} \varepsilon_{c}=0.1747\varepsilon_{p}\\ \varepsilon_{p}=0.03811{\dot{\varepsilon }}^{0.1791}\exp\left(24201.59/RT\right)\\ \varepsilon_{0.5}=0.03432{\dot{\varepsilon }}^{0.1376}\exp\left(23059.65/RT\right)\\ X_{\mathrm{drx}}=1-\exp\left[-1.69{\left(\frac{\varepsilon -\varepsilon_{c}}{\varepsilon_{0.5}}\right)}^{1.7959}\right] \end{array}\right.$$

#### 3.2.3. The Grain Size Model of DRX

**Figure 12:**
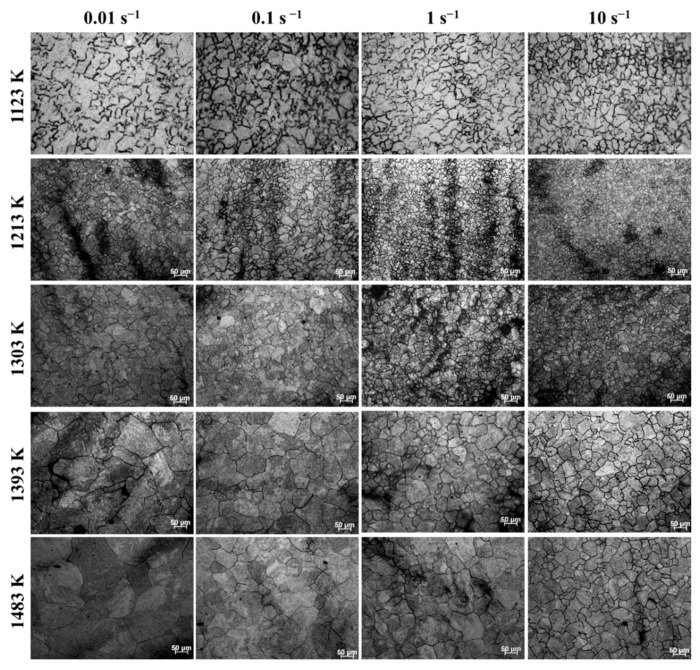
Microstructures after compression under temperatures of 1123–1483 K and strain rates of 0.01–10 s^−1^.

Figure 12 exhibits the microstructures after compression under temperatures of 1123–1483 K and strain rates of 0.01–10 s^−1^. Apparently, the microstructures after compression strongly depend on temperature and strain rate. With the increase of strain rate, there are abundant equiaxed and refined grains formed in the microstructures. In addition, the microstructures become more homogeneous with temperature increasing. The observation results indicate that DRX has occurred during the isothermal compression process of SAE 5137H steel. Furthermore, the average grain size of microstructures in Figure 12 was measured, and their values were listed in Table 3. It is apparently found that the average grain size after compression decreases with increasing strain rate and decreasing temperature. From the results of average grain size, it also can be seen that the microstructures after compression are refined under most conditions when comparing with the initial microstructures. 

In this work, the Sellars model was used to characterize the evolution of grain size during the hot deformation process of SAE 5137H steel, in which DRX grain size is strongly associated with initial grain size, temperature, and strain rate, as described in Equation (14) [25]:

(14) $$d_{drx}=a_{3}d_{0}^{n_{3}}{\dot{\varepsilon }}^{m_{3}}\exp(-Q_{3}/RT)$$

where $d_{\mathrm{drx}}$ is the size of recrystallized grains; $d_{0}$ is the initial grain size, i.e., 64.8 μm; *Q*_3_ is deformation activation energy; *a*_3_, *n*_3_ and *m*_3_ are material constants. 

Taking the natural logarithm on both sides of Equation (14) yields Equation (15):

(15) $$\ln d_{drx}=\ln a_{3}+n_{3}\ln d_{0}+m_{3}\ln\dot{\varepsilon }-\frac{Q_{3}}{RT}$$

**Figure 13:**
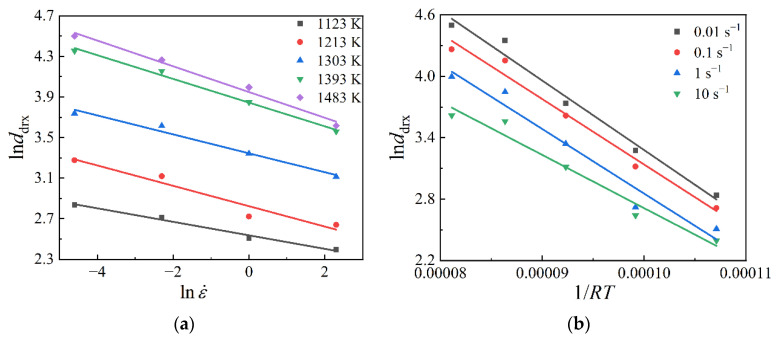
Relationships of (**a**) $\ln d_{\mathrm{drx}}$~$\ln\dot{\varepsilon }$ and (**b**) $\ln d_{\mathrm{drx}}$ ~1/RT.

As the effect of initial grain size has been overlooked in this work, i.e., *n*_3_ = 0, the material constants *m*_3_ and *Q*_3_ can be derived from Equation (15) that $m_{3}=\partial \ln d_{\mathrm{drx}}/\partial \ln\dot{\varepsilon }$ and $Q_{3}=-R\partial \ln d_{\mathrm{drx}}/\partial (1/T)$. The relationships of $\ln d_{\mathrm{drx}}$~$\ln\dot{\varepsilon }$ and $\ln d_{\mathrm{drx}}$~1/RT were fitted linearly as Figure 13. Then, *m*_3_ and *Q*_3_ were evaluated as –0.10067 and –61735.26, respectively. By substituting the calculated results of *m*_3_ and *Q*_3_ into Equation (14), the value of *a*_3_ was figured out as 8594.31. 

Therefore, the grain size model of DRX for SAE 5137H steel can be summarized as Equation (16):

(16) $$d_{drx}=8594.31{\dot{\varepsilon }}^{-0.10067}\exp\left(-61735.26/RT\right)$$

### 3.3. Description of DRX Behaviors

It is well-accepted that the resistance heating isothermal compression can be summarized as an electrical-thermal-mechanical multi-field issue [28,29]. In order to describe the evolution of DRX behaviors in the hot compression process of SAE 5137H steel, the obtained true stress–strain data and established DRX kinetics models were programed into finite element codes, and then the FE model was developed on the DEFORM-3D platform. In this FE model, the workpiece was simplified as a cylindrical specimen with 10 mm in diameter and 12 mm in height. The workpiece was defined as a plastic body without elastic deformation, and the two anvils were set as rigid bodies. The friction between workpiece and anvils was assumed as shear type, and the friction coefficient was taken as 0.3. The heat transfer coefficient between workpiece and anvils was set as 0.033. The initial grain size of workpiece was set as 64.8 μm. The velocity of compressing anvil can be calculated from Equation (17) [28]. Based on the developed FE model, the hot compression processes in agreement with the experimental conditions were simulated, and the DRX behaviors were discussed:

(17) $$v=l_{0}\dot{\varepsilon }\exp(-\dot{\varepsilon }t)$$

where v is the velocity of compressing anvil; $\dot{\varepsilon }$ is the proposed strain rate; *t* is time; *l*_0_ is the initial height of workpiece.

**Figure 14:**
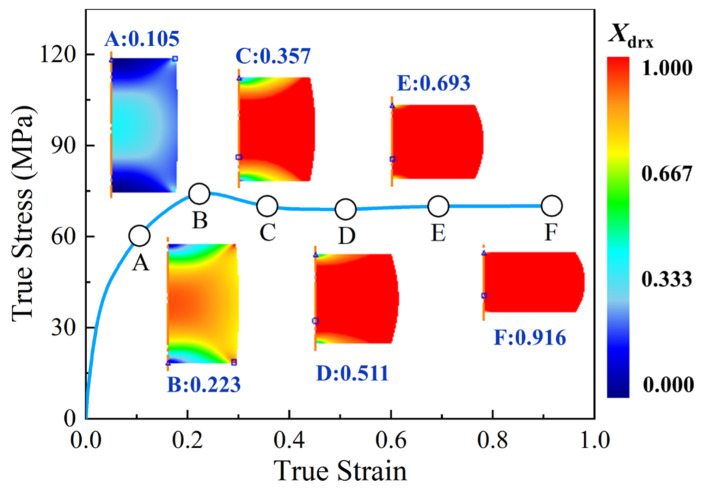
Evolution and distributions of DRX volume fraction of the specimen compressed to different true strains under the temperature of 1303 K and strain rate of 0.1 s^−1^.

When the specimen deformed at 1303 K and 0.1 s^−1^, the distributions of DRX volume fraction at different true strains were demonstrated in Figure 14, in which the true strains of 0.105, 0.223, 0.357, 0.511, 0.693, and 0.916 correspond to height reductions of 10%, 20%, 30%, 40%, 50%, and 60%, respectively. In Figure 14, it can be observed that, when the true strain increases from 0.105 to 0.511 (point A to point D), DRX volume fraction increases appreciably. When the true strain exceeds 0.511 (point D to point F), DRX occurs completely, DRX volume fraction is changed insignificantly with the continuous increase of true strain. Moreover, it can also be noted that the distribution of DRX volume fraction is not uniform. At the center of a deformed specimen, DRX volume fraction is the maximum, while it is relatively small at the center of upper and lower end-surfaces of the specimen. This is owing to the fact that the nonlinearly interactions of temperature field, strain rate field, and strain field are distributed non-homogeneously, which directly affects the microstructure evolution in the hot deformation process of materials [5,29]. 

**Figure 15:**
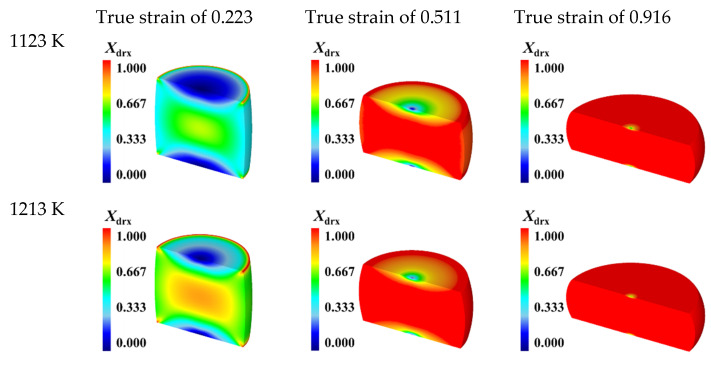

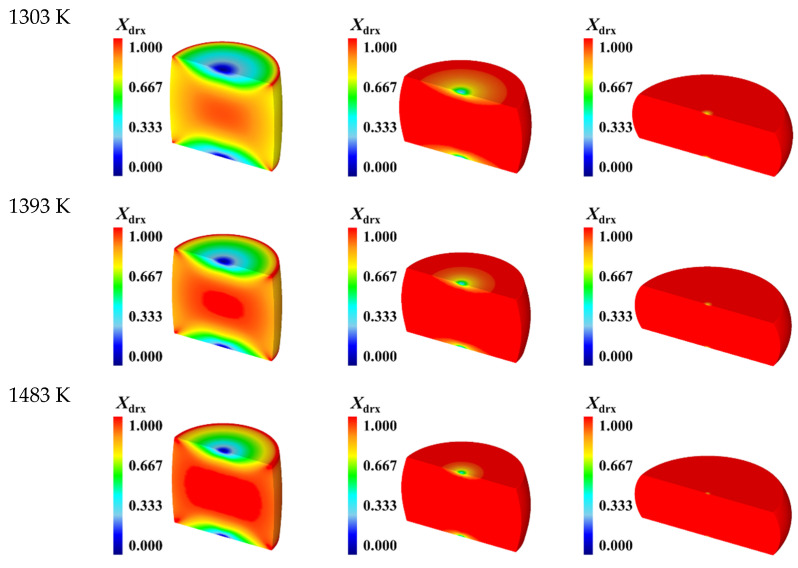
DRX volume fraction distributions of the specimens compressed to different true strains under the strain rate of 0.1 s^−1^ and different temperatures of 1123–1483 K.

**Figure 16:**
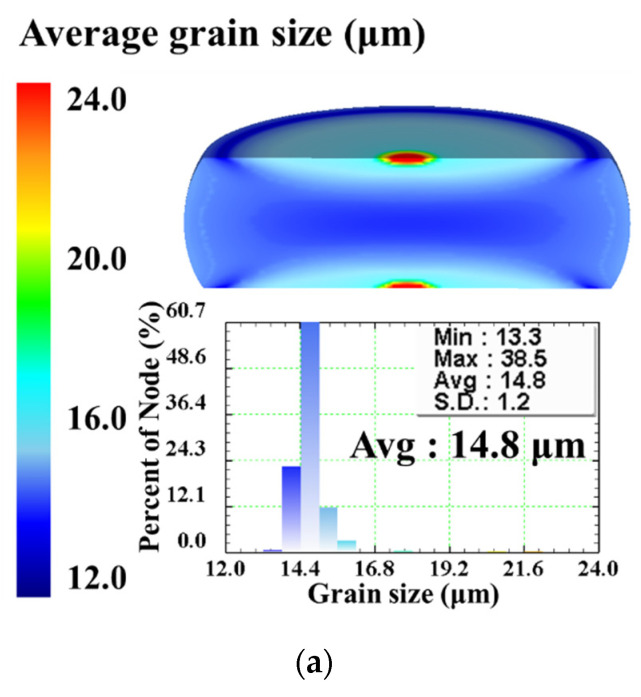

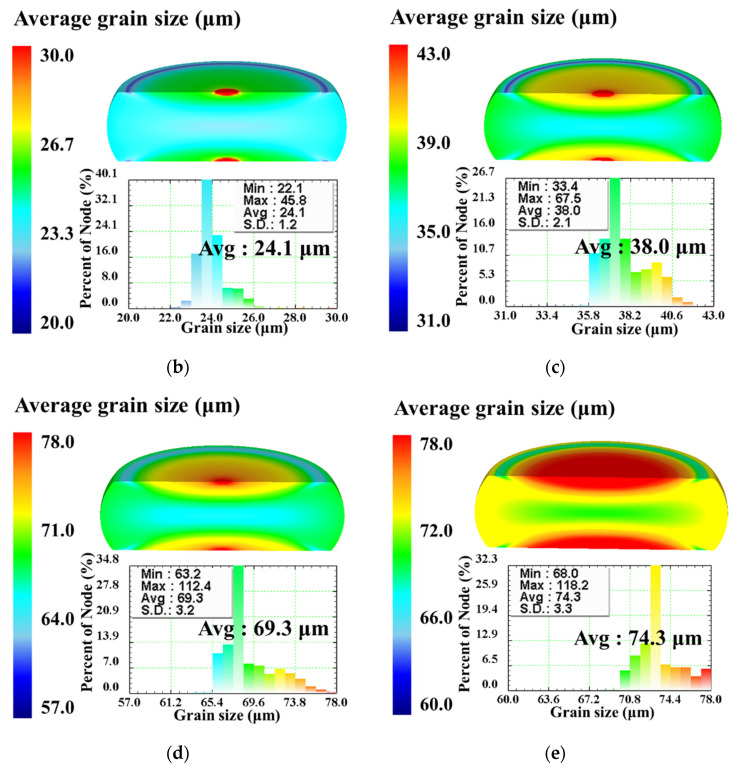
Grain size distributions of the specimens compressed to the true strain of 0.916 under the strain rate of 0.1 s^−1^ and different temperatures: (**a**) 1123 K, (**b**) 1213 K, (**c**) 1303 K, (**d**) 1393 K, (**e**) 1483 K.

Figure 15 exhibits the DRX volume fraction distributions of the specimens isothermally compressed to different true strains of 0.223, 0.511, and 0.916 under the constant strain rate of 0.1 s^−1^ and different deformation temperatures of 1123K, 1213 K, 1303 K, 1393 K, and 1483 K. It is evident that, for a fixed true strain, the distribution of DRX volume fraction at any given temperature is similar to each other. From the center region to the outer edge, DRX volume fraction gradually decreases, and its maximum value appears at the center region of the deformed specimen. As true strain increases from 0.223 to 0.916, the DRX volume fraction in all region of specimen increases. By comparing the distribution of DRX volume fraction under different temperatures, it is shown that the volume fraction of DRX grains increases with increasing temperature. In addition, it is worth noting that, at higher temperature, DRX volume fraction at the center region of specimen has reached the maximal value at a relatively small true strain. This is owing to the fact that higher temperature can provide more activation energy for the nucleation of DRX grains. Hence, DRX occurs more easily and more completely at elevated temperature. The distributions of grain size at the end of hot compression (the true strain of 0.916) under the constant strain rate of 0.1 s^−1^ and different temperatures of 1123K, 1213 K, 1303 K, 1393 K, and 1483 K were demonstrated in Figure 16a–e, respectively. It is noticed that the distribution of grain size shows an opposite variation tendency with DRX volume fraction distribution. From the center region to the outer edge, grain size gradually increases. At the center region of the deformed specimen, grain size reaches the minimum value, while its maximum value appears at the center of the upper and lower end-surfaces. An obvious feature can be observed from Figure 16a–e is that the distributions of grain size are not uniform in the deformed specimens under different temperatures. The standard deviation (SD) of grain size distribution gradually increases as the temperature increases. It indicates that the inhomogeneous degree of grain size increases with temperature increasing. Under the temperatures of 1123 K, 1213 K, 1303 K, 1393 K, and 1483 K, the values of average grain size (denoted as “Avg.” in Figure 16) are calculated as 14.8 μm, 24.1 μm, 38.0 μm, 69.3 μm, and 74.3 μm, respectively. It reveals that grain size increases with the increase of temperature. The reasons accounting for this phenomenon are as follows. As we all know, grain size is determined by the comprehensive action of grain refinement induced by DRX and grain coarsening resulted from grain growth [9,29]. At higher temperatures, the occurrence of DRX can be significantly promoted, resulting in the increase of DRX volume fraction—while, at the same time, the grain boundary migration rate is higher at elevated temperatures. The mechanism of grain growth plays a dominant role in the evolution of grain size, thus provoking grain coarsening. From the simulation results in Figure 15 and Figure 16, it can be concluded that DRX volume fraction and grain size increase with temperature increasing.

**Figure 17:**
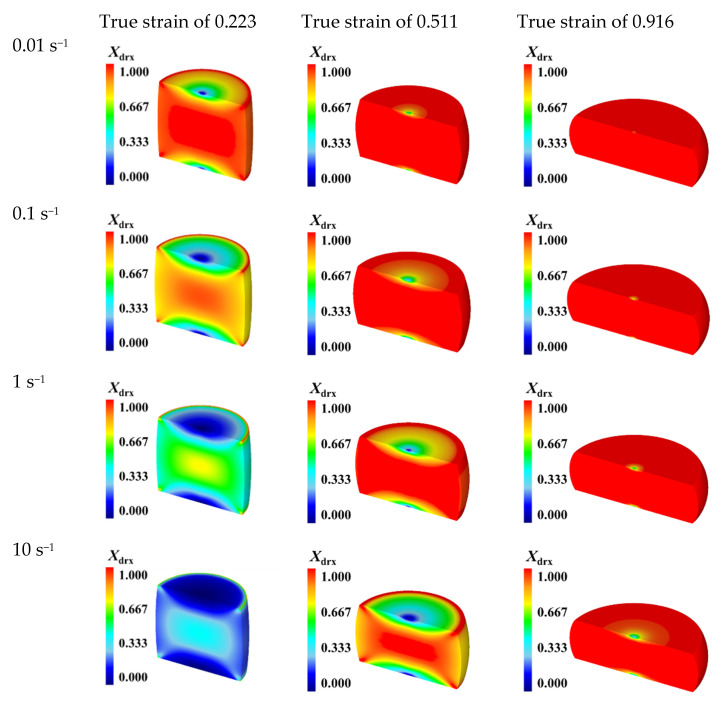
DRX volume fraction distributions of the specimens compressed to different true strains under the temperature of 1303 K and different strain rates of 0.01–10 s^−1^.

**Figure 18:**
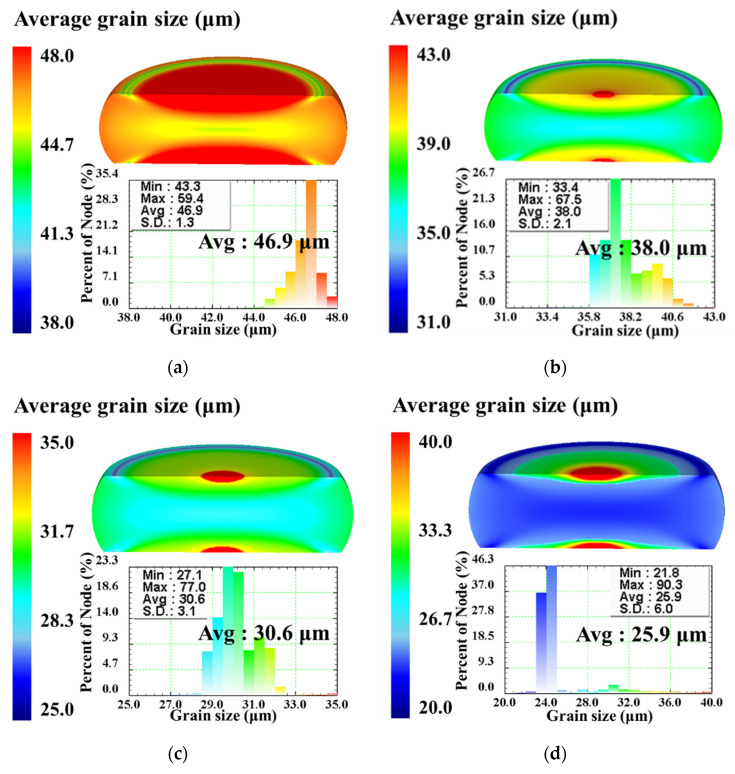
Grain size distributions of the specimens compressed to the true strain of 0.916 under the temperature of 1303 K and different strain rates: (**a**) 0.01 s^−1^, (**b**) 0.1 s^−1^, (**c**) 1 s^−1^, (**d**) 10 s^−1^.

**Figure 19:**
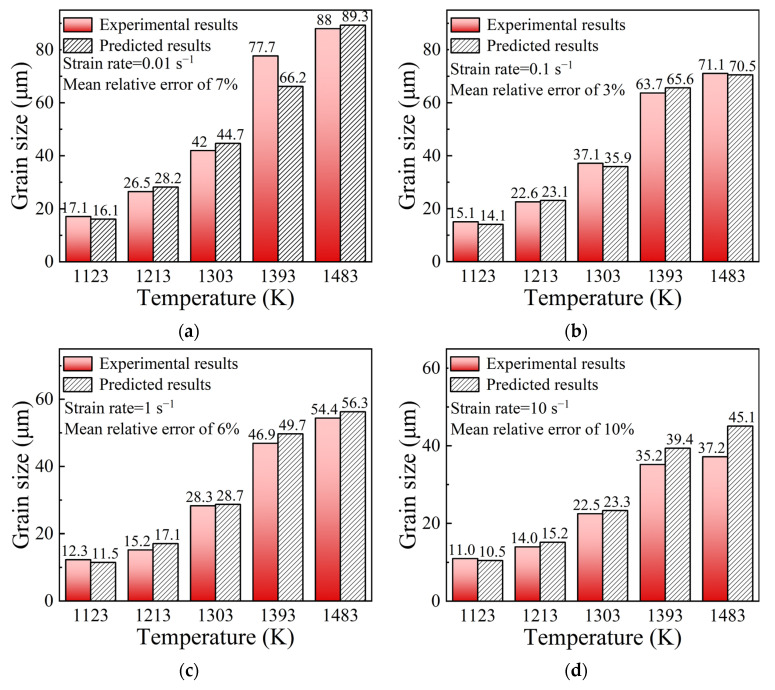
Comparisons of grain size between experimental results and predicted ones from DRX kinetics models under temperatures of 1123–1483 K and different strain rates: (**a**) 0.01 s^−1^, (**b**) 0.1 s^−1^, (**c**) 1 s^−1^, (**d**) 10 s^−1^.

Figure 17 illustrates the distributions of DRX volume fraction of the specimens compressed to different true strains of 0.223, 0.511, and 0.916 under the constant temperature of 1303 K and different strain rates of 0.01 s^−1^, 0.1 s^−1^, 1 s^−1^, and 10 s^−1^. Comparing Figure 17 with Figure 15, it can be seen clearly that, for a constant true strain, DRX volume fraction exhibits the similar distribution under diverse temperatures and strain rates. The distributions of DRX volume fraction at different strain rates are also inhomogeneous in the deformed specimens. At the true strain of 0.223, a significant difference on the distributions of DRX volume fraction can be observed under the strain rates of 0.01 s^−1^ and 10 s^−1^. Especially, for the center region of specimen, the DRX volume fraction at the strain rate of 0.01 s^−1^ is much higher than that in the strain rate of 10 s^−1^. In addition, this difference is decreased when the true strain exceeds 0.511, since DRX occurs completely with the continuous increase of strain. The comparisons of the distributions of DRX volume fraction under different strain rates indicate that DRX volume fraction increases with strain rate decreasing, and, meanwhile, for a fixed true strain, the center region of specimen with relatively high DRX volume fraction also increases as well. This attributes to the fact that the lower strain rate can provide enough time for the DRX process, and then DRX occurs more completely. Figure 18a–d display the distributions of grain size at the end of hot compression under the constant temperature of 1303 K and different strain rates of 0.01 s^−1^, 0.1 s^−1^, 1 s^−1^, and 10 s^−1^, respectively. It is noted that the distributions of grain size in the deformed specimens exhibit the similar tendency under different strain rates, and all distributions are inhomogeneous. The standard deviation (SD) of grain size distribution increases as the strain rate increases, which implies that the degree of deformation inhomogeneity increases with the strain rate increasing. Under the strain rate of 0.01 s^−1^, 0.1 s^−1^, 1 s^−1^, and 10 s^−1^, the values of average grain size (denoted as “Avg.” in Figure 18) are calculated as 46.9 μm, 38.0 μm, 30.6 μm, and 25.9 μm, respectively. It indicates that grain size becomes finer with strain rate increasing. On the one hand, the dislocation generation rate and dislocation density increase with the increase of strain rate. There are more deformation energies stored in the deformed SAE 5137H steel, which promotes the nucleation of DRX grains. On the other hand, there is no sufficient time for grain growth, resulting in finer grain size at a higher strain rate. Based on the simulation results in Figure 17 and Figure 18, it can be summarized that DRX volume fraction and grain size increase with the strain rate decreasing:

In order to examine the predictive ability of the multi-field and multi-scale coupling FE model embedded with DRX kinetics models, for the center of specimens marked “X” in Figure 3, the values of grain size were computed from simulation results. The comparisons of grain size between experimental results and predicted ones were performed, as shown in Figure 19. The mean relative error is calculated as 6.5%, which indicates a good agreement between the predicted and experimental results. It strongly confirms that the established DRX kinetics models can be successfully incorporated into the FE model to describe the DRX behaviors of SAE 5137H steel in the hot deformation process. 

## 4. Description of Grain Evolution Mechanisms Involving DRX

### 4.1. Meso-Scale Modeling of Grain Evolution Mechanisms Involving DRX

#### 4.1.1. Modeling on Dislocation Evolution

During a hot deformation process, the evolution of dislocation density is associated with WH and softening behaviors. WH causes the increase of dislocation density, while DRV and DRX softening behaviors result in the decrease of dislocation density. The dislocation density can be expressed by the Laasroui–Jonas model, as shown in Equation (18) [30,31,32]:

(18) $$d\rho_{i}=\left(h-r\rho_{i}\right)d\varepsilon -\rho_{i}d\varepsilon$$

where $\rho_{i}$ is the dislocation density of the *i*-th new grain. Here, the initial dislocation density is taken as 0.01 µm^−2^; *h* and *r* are hardening coefficient and recovery coefficient, which can by calculated by Equation (19) and Equation (20), respectively:

(19) $$h=h_{0}{\left(\frac{\dot{\varepsilon }}{{\dot{\varepsilon }}_{0}}\right)}^{-m}\exp\left(\frac{-mQ}{RT}\right)$$

(20) $$r=r_{0}{\left(\frac{\dot{\varepsilon }}{{\dot{\varepsilon }}_{0}}\right)}^{-m}\exp\left(\frac{-mQ}{RT}\right)$$

where $h_{0}$ is hardening constant; $r_{0}$ is recovery constant; ${\dot{\varepsilon }}_{0}$ is strain rate calibration constant taken as 1; *Q* is apparent activation energy; *m* is strain-rate sensitivity. 

From Ref. [33], the recovery coefficient *r* is strongly related to strain hardening rate *θ* and flow stress, and the relationship can be expressed as Equation (21):

(21) $$2\sigma \theta =r\sigma_{\mathrm{sat}}^{2}-r\sigma^{2}$$

where $\sigma_{\mathrm{sat}}$ is steady state stress in the ideal DRV-type stress–strain curves, and its values were given in Table 4. 

**Figure 20:**
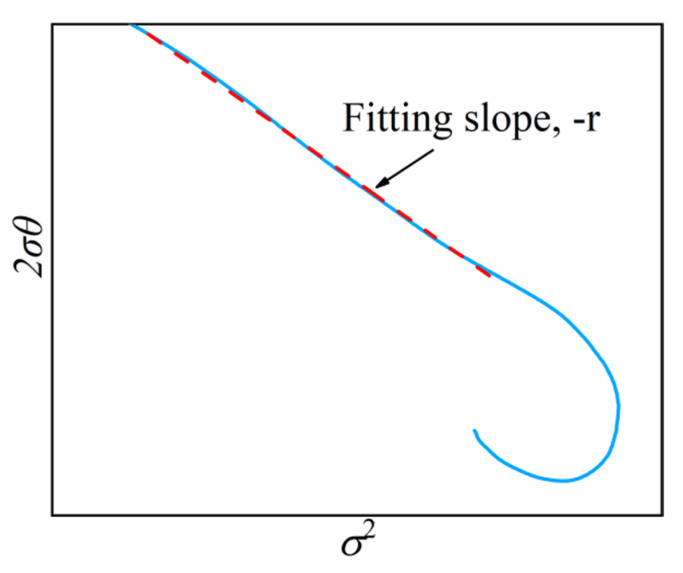
Fitting slope of 2σθ~$\sigma^{2}$ curve.

In Equation (21), it can be derived that $r=-\frac{d\left(2\sigma \theta \right)}{d\left(\sigma^{2}\right)}$, the slope of 2σθ~$\sigma^{2}$ curve in Figure 20 is the approximate slope *k*, then *r* = −*k*. The values of *r* were given in Table 5.

Take the natural logarithm on both sides of Equation (20) and give Equation (22):

(22) $$\ln r=\ln r_{0}-m\ln\dot{\varepsilon }-mQ/RT$$

Substituting the obtained values of *r* into Equation (20), the parameters can be calculated by multivariate linear fitting, *m* = 0.06594, *Q* = 382,301.89 J·mol^−1^, *r*_0_ = 159.74.

According to Ref. [32], the hardening coefficient *h* can be calculated by Equation (23):

(23) $$h=\frac{r\sigma_{\mathrm{sat}}^{2}}{{\left(\alpha \mu b\right)}^{2}}$$

where *µ* is shear modulus; *b* is Burgers vector; and *α* is Taylor factor taken as 1. 

Substituting the obtained values of *r* and $\sigma_{\mathrm{sat}}$ into Equation (23), the values of *h* can be calculated, as listed in Table 6. Then, substituting the above parameters into Equation (19), *h*_0_ can be obtained as 56.33.

Here, the Goetz recovery model was employed to describe the influence of recovery mechanism on the evolution of dislocation, as expressed in Equation (24) [29]:

(24) $$N_{r}=\frac{{\left[\left(\#rows\right)\times \left(\#columns\right)\times \sqrt{2}\right]}^{2}}{K}h{\left(d\varepsilon \right)}^{\left(1-2m\right)}$$

where *K* is a constant taken as 6030; *N*_r_ is the amount of cells in which DRX occurs; “#*rows*” and “#*columns*” are the number of rows and columns of discrete lattice points, respectively.

#### 4.1.2. Grain Nucleation and Growth Models

It is well-known that dislocation density increases continuously as strain increases. When the dislocation density reaches a critical value, DRX grains first nucleate at the grain boundary. The relationship between nucleation rate $\dot{n}$ and strain rate can be described as Equation (25) [18]:

(25) $$\dot{n}=C{\dot{\varepsilon }}^{l}$$

where *C* and *l* are material constants taken as 0.9 and 200, respectively.

The dislocation density difference between new grains and original grains provides the driving force for grain growth, and the new grains can grow continuously until the dislocation density difference is zero. The driving force *F* can be expressed as Equation (26) [34]:

(26) $$F=4\pi r_{i}^{2}\tau \left(\rho_{m}-\rho_{i}\right)-8\pi r_{i}\gamma$$

where $r_{i}$ is DRX grain size; τ is the energy of dislocation line; $\rho_{i}$ is the dislocation density of new DRX grains; $\rho_{m}$ is the dislocation density of matrix; γ is grain boundary energy, which can be calculated by Equation (27) [15]:

(27) $$\gamma =\left\{\begin{array}{ll} \gamma_{m} & \theta_{i}\geq 15^{{}^{\circ}}\\ \gamma_{m}\frac{\theta_{i}}{\theta_{m}}\left(1-\ln(\frac{\theta_{i}}{\theta_{m}})\right) & \theta_{i}<15^{{}^{\circ}} \end{array}\right.$$

where $\theta_{i}$ is the misorientation between two adjacent grains; $\theta_{m}$ is the critical misorientation of high angle grain boundary, and it is usually taken as 15°; $\gamma_{m}$ is the energy of high angle grain boundary, which can be calculated from Equation (28):

(28) $$\gamma_{m}=\frac{\mu b\theta_{m}}{4\pi (1-\nu )}$$

where ν is Poisson ratio.

### 4.2. Description of Grain Evolution Mechanisms in the Hot Deformation Process

In order to describe the grain morphology evolution process in the hot deformation process of SAE 5137H steel, the solved dislocation evolution models, grain nucleation, and growth models were embedded into the FE model in hot deformation process, and then the FE model embedded with CA models was developed. In this model, a two-dimensional CA model with a simulation area of 800 μm × 800 μm was constructed, and the periodic Moore’s neighboring rule was applied in it. The initial grain morphology was generated through the following steps [35]. According to the measured initial grain size, a certain amount of cells in the grid framework was randomly selected as the nucleation sites for initial grains. The number of nucleation sites can be determined by Equation (29). Random grain orientations represented by integer values from 1 to 60 were assigned to the selected cells. Then, these seed cells begin to grow towards the surroundings with a certain velocity. Once two grains with different orientations collide, they will stop growing at the adjacent boundaries, while the other portion of the boundaries will continue to grow. If two grains have the same orientation, they will merge into one grain. As time goes by, the grains continuously grow until the whole calculation domain is filled up:

(29) $$N=\frac{4S}{\pi d_{0}{}^{2}}$$

where *N* is the number of nucleation sites; *S* is the simulation area.

**Figure 21:**
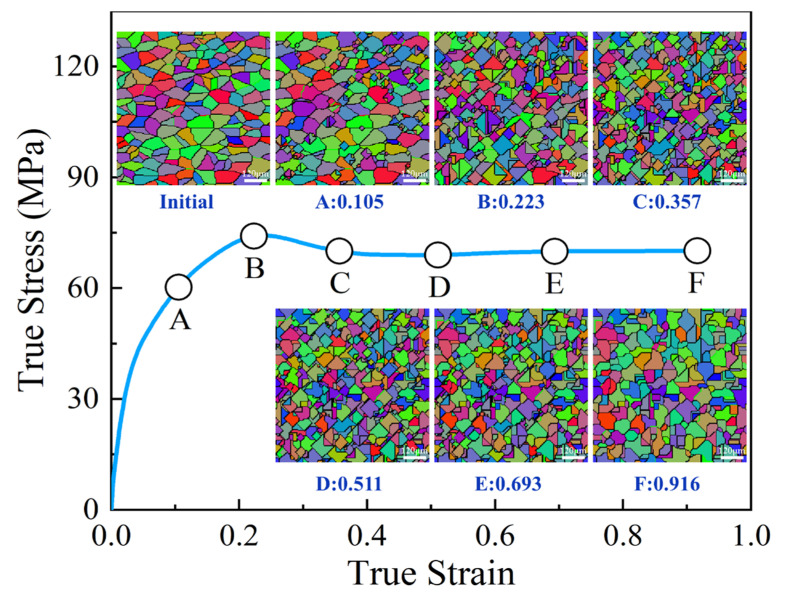
Evolution process of grain morphology under the temperature of 1303 K and strain rate of 0.1 s^−1^.

For the temperatures of 1123–1483 K and strain rates of 0.01–10 s^−1^, the grain morphology evolution during the hot deformation process of SAE 5137H steel was simulated numerically. Then, the grain evolution mechanisms were uncovered. Figure 21 shows the evolution process of grain morphology in the center of deformed specimen under the temperature of 1303 K and strain rate of 0.1 s^−1^. In Figure 21, it is obvious that the recrystallized grains appear near the initial grain boundaries when the deformation degree exceeds a certain value. As the deformation increases continuously, the recrystallized grains grow up by devouring the matrix grains till the entire selected area achieves complete recrystallization. Combining Figure 21 with Figure 14, it is found that, when the true strain increases from 0.105 to 0.357 (point A to point C), DRX volume fraction increases, and more and more recrystallized grains are formed. When the strain increases from 0.511 to 0.916 (point D to point F), dislocation density continues to multiply as deformation increases, and, meanwhile, the new recrystallized grains generate again, resulting in the annihilation of dislocation. This also explains why flow stress finally keeps a steady value under the dynamic equilibrium between work hardening and dynamic softening. Furthermore, it can also be observed that the deformed microstructures tend to be more uniform when the true strain exceeds 0.511. Compared with the initial microstructures, the microstructures after compression are refined and composed of equiaxed DRX grains.

**Figure 22:**
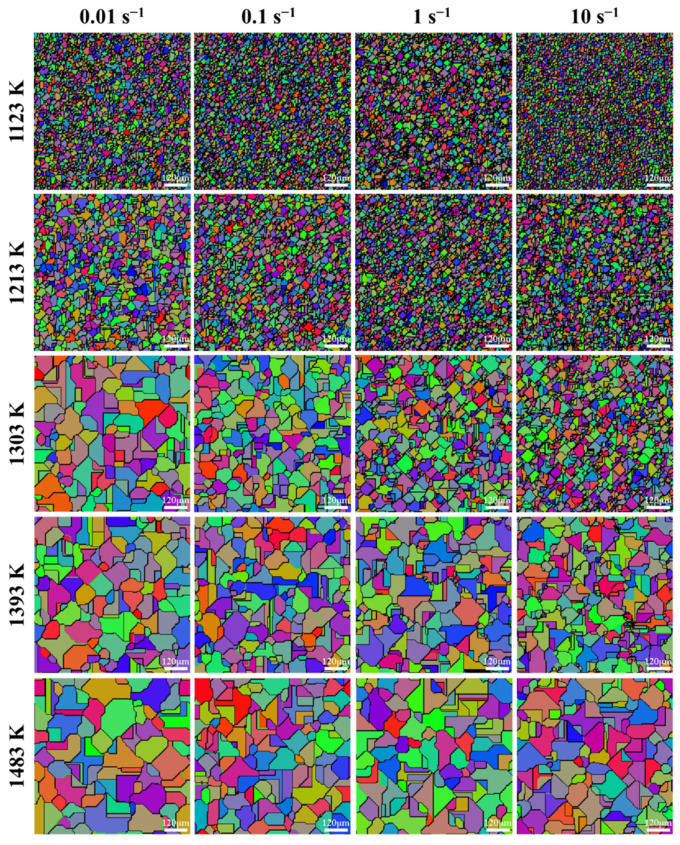
Grain morphology of the specimens compressed to a fixed true strain of 0.916 under temperatures of 1123–1483 K and strain rates of 0.01–10 s^−1^.

As analyzed in Section 3.3, DRX volume fraction and grain size are closely associated with deformation temperature and strain rate. In order to reveal the effects of strain rate and temperature on grain morphology evolution, the hot compression processes under the temperatures of 1123–1483 K and strain rates of 0.01–10 s^−1^ were simulated by the same way. Figure 22 depicts the grain morphology at the end of hot compression (the true strain of 0.916) under various temperatures and strain rates. From Figure 22, it can be easily found that, for a constant temperature, more and more DRX grains are formed with the increase of strain rate. On the one hand, the dislocation generation rate, the dislocation density, and nucleation sites in the deformed microstructures increase with strain rate increasing. Then, for the case of high strain rate, there is sufficient deformation energy accumulated in the deformed specimen, which contributes to the occurrence of the DRX process. Consequently, more sub-strains can be formed in the initial microstructures when the strain rate is relatively high, which increases nuclei per unit volume of the grains [36]. On the other hand, higher strain rate provides a shorter time for grain growth after complete recrystallization, eventually leading to grain refinement after compression. These mechanisms cause finer grains in the microstructures at a higher strain rate. From the simulation results in Figure 22, it can also be seen that, for a constant strain rate, grain size increases remarkably with the increase of temperature, and the microstructures tend to be more uniform. Tracking its causes, there is more activation energy for the nucleation of DRX grains at elevated temperature, thereby facilitating the annihilation of dislocation. Meanwhile, higher temperature can improve grain boundary migration rate. The sub-grains with high dislocation density are easier to transform into the dislocation-free DRX grains. Thus, DRX happens more completely with temperature increasing (as shown in Figure 15). Nevertheless, it is worth emphasizing that, due to such a high grain boundary migration rate, DRX grains are more likely to grow up under higher temperature. Thus, the grain size in the deformed microstructures becomes coarser as temperature increases. Furthermore, it can be seen from Figure 22 that there are some straight grain boundaries existing in the microstructures with larger grain size. The straight grain boundaries may come from the inherent features of CA simulation. As we all know, in a CA simulation, the grain morphology is composed of a large number of discrete cells, and the cells are just like the pixels of a picture. The grain boundary with small curvature usually looks like a straight line in the CA simulation graphs. When the specimen is deformed at higher temperature and lower strain rate, DRX grains grow up, resulting in the reduction of boundary curvature. Correspondingly, some portions of grain boundary exhibit the characteristic of a straight line in the CA simulation graphs. In addition, these characteristics are more obvious in the microstructures with coarse grains. 

**Figure 23:**
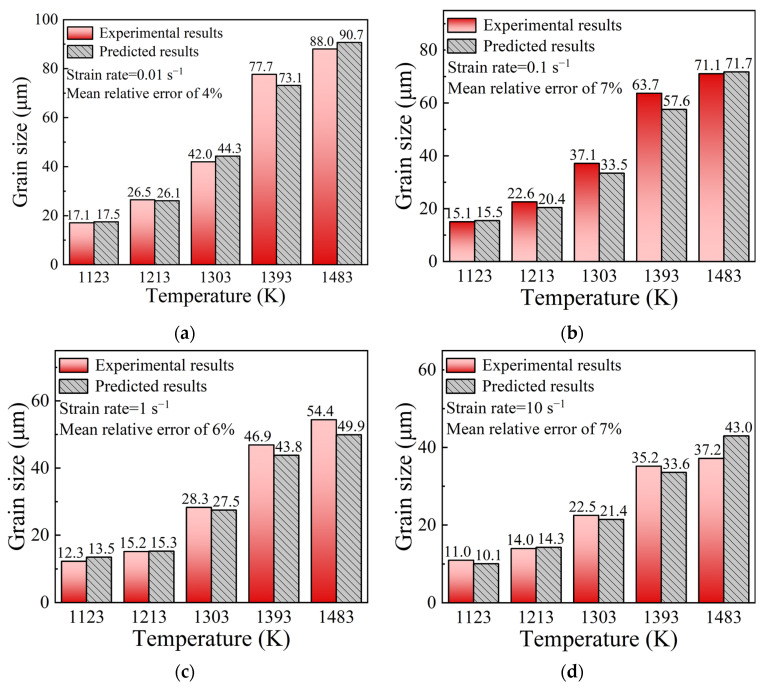
Comparisons of grain size between experimental results and predicted ones from CA models under temperatures of 1123–1483 K and different strain rates: (**a**) 0.01 s^−1^, (**b**) 0.1 s^−1^, (**c**) 1 s^−1^, (**d**) 10 s^−1^.

In order to validate the simulation results, for the center of specimens marked “X” in Figure 3, the grain size in the microstructures (Figure 22) at different temperatures and strain rates were statistically analyzed. Figure 23 shows the comparisons of grain size between experimental results and predicted ones. The mean relative error is calculated as 6.0%, which indicates that the predicted results are consistent with experimental results. It strongly confirms that the developed FE model embedded with CA models can well describe the grain morphology evolution of SAE 5137H steel in the hot deformation process. 

## 5. Conclusions

The dynamic recrystallization behaviors and grain evolution mechanisms during the hot forming process of SAE 5137H steel were investigated by hot compression experiments under temperatures of 1123–1483 K and strain rates of 0.01–10 s^−1^. The main conclusions drawn from this work are as follows:

(1) The DRX kinetics and grain size models in the hot deformation process of SAE 5137H steel were established based on the stress–strain data and microstructure observation results. The solved models are as follows:

$$\left\{\begin{array}{l} \varepsilon_{c}=0.1747\varepsilon_{p}\\ \varepsilon_{p}=0.03811{\dot{\varepsilon }}^{0.1791}\exp\left(24201.59/RT\right)\\ \varepsilon_{0.5}=0.03432{\dot{\varepsilon }}^{0.1376}\exp\left(23059.65/RT\right)\\ X_{\mathrm{drx}}=1-\exp\left[-1.69{\left(\frac{\varepsilon -\varepsilon_{c}}{\varepsilon_{0.5}}\right)}^{1.7959}\right]\\ d_{drx}=8594.31{\dot{\varepsilon }}^{-0.10067}\exp\left(-61735.26/RT\right) \end{array}\right.$$

(2) The multi-field and multi-scale coupling FE model for the hot compression process was developed to describe the DRX behaviors and grain size evolution of SAE 5137H steel. The simulation results show that, with increase of strain, DRX volume fraction increases, and grain size is refined gradually. For a constant strain rate, DRX volume fraction and grain size increase with temperature increasing. For a constant temperature, DRX volume fraction and grain size decrease with strain rate increasing. 

(3) The CA models of SAE 5137H steel were established and embedded into the FE model to reveal the evolution process of grain morphology in the hot compression process. The results indicate that the recrystallized grains are easier to nucleate at the initial grain boundaries. With strain increasing, the recrystallized grains grow up by devouring the matrix grains until DRX occurs completely. The microstructures after compression are composed of equiaxed DRX grains. 

(4) The comparisons of grain size between experimental results and simulation results were performed. The mean relative errors between experimental results and predicted results from DRX kinetics models, between experimental results and predicted results from CA models were evaluated as 6.5% and 6.0%, respectively. This result indicates that the developed FE model can well describe the microstructure evolution in the hot forming process of SAE 5137H steel.

## Figures and Tables

**Table 1:** Chemical composition (wt%) of studied SAE 5137H steel.

Element	C	Mn	Si	S	P	Cr	Mo	Ni
Content	0.38	1.19	0.28	0.025	0.015	1.23	0.042	0.11

**Table 2:** Values of $\varepsilon_{c}$ and $\varepsilon_{p}$ under temperatures of 1123–1483 K and strain rates of 0.01–10 s^−1^.

Temperature (K)	Strain Rate (s^−1^)							
	0.01		0.1		1		10	
	$\varepsilon_{c}$	$\varepsilon_{p}$	$\varepsilon_{c}$	$\varepsilon_{p}$	$\varepsilon_{c}$	$\varepsilon_{p}$	$\varepsilon_{c}$	$\varepsilon_{p}$
1123	0.0921	0.2320	0.1321	0.3512	0.1472	0.5233	0.1353	0.5548
1213	0.0817	0.1943	0.0960	0.3256	0.1228	0.4958	0.1170	0.5535
1303	0.0682	0.1539	0.0749	0.2376	0.1354	0.3479	0.1209	0.5249
1393	0.0560	0.1298	0.0468	0.1793	0.1108	0.3353	0.1141	0.5004
1483	0.0442	0.1057	0.0561	0.1439	0.1016	0.2772	0.1152	0.4988

**Table 3:** Average grain size and corresponding standard deviation of SAE 5137H steel under temperatures of 1123–1483 K and strain rates of 0.01–10 s^−1^.

Temperature (K)	Strain Rate (s^−1^)			
	0.01	0.1	1	10
1123	(17.1 ± 3.0) μm	(15.1 ± 2.6) μm	(12.3 ± 2.3) μm	(11.0 ± 2.2) μm
1213	(26.5 ± 3.6) μm	(22.6 ± 3.2) μm	(15.2 ± 2.5) μm	(14.0 ± 2.3) μm
1303	(42.0 ± 4.6) μm	(37.1 ± 4.0) μm	(28.3 ± 3.6) μm	(22.5 ± 3.1) μm
1393	(77.7 ± 6.3) μm	(63.7 ± 5.6) μm	(46.9 ± 4.1) μm	(35.2 ± 4.4) μm
1483	(88.0 ± 5.5) μm	(71.1 ± 5.2) μm	(54.4 ± 4.0) μm	(37.2 ± 4.3) μm

**Table 4:** Values of $\sigma_{\mathrm{sat}}$ under temperatures of 1123–1483 K and strain rates of 0.01–10 s^−1^.

Temperature (K)	Strain Rate (s^−1^)			
	0.01	0.1	1	10
1123	124.12	173.02	233.75	343.69
1213	79.14	121.63	169.01	290.10
1303	59.45	100.29	100.60	169.96
1393	45.98	69.30	90.75	136.12
1483	24.79	48.79	59.76	97.00

**Table 5:** Values of *r* under temperatures of 1123–1483 K and strain rates of 0.01–10 s^−1^.

Temperature (K)	Strain Rate (s^−1^)			
	0.01	0.1	1	10
1123	14.14	12.46	11.13	10.40
1213	18.20	15.28	12.56	11.06
1303	21.65	18.08	14.26	12.81
1393	24.20	19.50	16.64	14.66
1483	29.74	25.00	22.48	19.36

**Table 6:** Values of *h* under temperatures of 1123–1483 K and strain rates of 0.01–10 s^−1^.

Temperature (K)	Strain Rate (s^−1^)			
	0.01	0.1	1	10
1123	520.81	891.82	1453.93	2937.06
1213	272.53	540.31	857.65	2223.80
1303	182.94	434.78	345.03	884.68
1393	122.30	223.88	327.65	649.42
1483	43.69	142.26	191.94	435.51

## Data Availability

Not applicable.
